# Mechanical and Morphological Assessment of an Innovative Textile for Patient Positioning Applications: Comparison to Two Standard Bandage Systems

**DOI:** 10.3390/ma14061508

**Published:** 2021-03-19

**Authors:** David Putzer, Dietmar Dammerer, Débora Coraça-Huber, Johannes Pallua, Werner Schmölz, Michael Nogler

**Affiliations:** 1 Experimental Orthopedics, Department of Orthopedic and Trauma Surgery, Medical University Innsbruck, 6020 Innsbruck, Austria; debora.coraca-huber@i-med.ac.at (D.C.-H.); michael.nogler@i-med.ac.at (M.N.); 2 Department of Orthopedics and Trauma Surgery, Medical University of Innsbruck, 6020 Innsbruck, Austria; dietmar.dammerer@i-med.ac.at (D.D.); johannes.pallua@i-med.ac.at (J.P.); werner.schmoelz@i-med.ac.at (W.S.)

**Keywords:** patient positioning system, test methods for textiles, stiffness of bandages, bandage systems, fixation of patients

## Abstract

In the healthcare environment, bandage systems are versatile medical devices to position and fix patients’ torsos or extremities. In this study, the mechanical and morphological properties of an innovative patient position system, iFix, were assessed and compared to two commercially available bandages. Morphological properties were investigated using a scanning electron microscope (SEM). The iFix bandage showed anisotropic mechanical properties, with a more rigid behavior in the longitudinal direction and a more elastic behavior in the transverse direction. This behavior results from the organization of the fibers visible in the SEM images. All three materials investigated in this study were able to support similar maximum loads. In cases where a rigid fixation of patient limbs or torso is necessary, the authors recommend the usage of iFix. In vivo studies should be carried out to prove safety in a surgical environment before its clinical usage.

## 1. Introduction

In healthcare environments, bandage systems are used to position and fix patients’ torsos or extremities on magnetic resonance tables, operation room tables or other holding devices to help healthcare professionals to carry out specific treatments. To our knowledge, only a few standards exist to classify bandage systems for patient positioning.

Partsch et al. provided methods for measuring the interface pressure and assessing the stiffness of a compression device in an individual patient [1]. The stiffness is defined by the increase in compression per centimeter increase in the circumference of the leg. This parameter characterizes a textile’s compliance, which has an essential role in the compression device’s performance.

The stiffness characterizes textile distensibility and varies with the position of the sensor, the shape of the body’s segment and the consistency of the underlying tissue. The stiffness indices should allow differentiation between short-stretch (extensibility lower than 100%) and long-stretch materials (extensibility more than 100%) [2]. Fabric stiffness under bending deformation influences the interface pressure that a compression garment can apply to a limb.

The measurement in vitro consists of measuring the exerted power required to distend the bandage and resulting stretch [2]. In vivo, the stiffness can be defined as the increase in compression per centimeter increase in the leg’s circumference (in the European Prestandard for Medical Compression Hosiery, Comité Européen de Normalisation), expressed in hectopascals per centimeter and/or millimeters of mercury per centimeter. Several studies have demonstrated that the difference between the sub-bandage pressure measured in standing and supine position is indirectly proportional to the bandage’s stretch length [2,3]. In a consensus meeting, proposals were made concerning methods for measuring the interface pressure and for assessing the stiffness of a compression device in an individual patient [1].

The static stiffness index (SSI) was defined as the difference between the interface pressure when standing and lying (mmHg) divided by 1 cm [4]. The SSI may be considered to characterize the in vivo stiffness of compression bandages, including multicomponent multilayer systems [4]. In the standing position, inelastic bandage systems will produce a higher sub-bandage pressure than elastic bandages, resulting in a higher SSI [4]. Bandage pressure should be assessed in vivo by measuring the interface pressure and calculation of the stiffness [5].

Measuring the dynamic behavior of medical compression hosiery was described by Stolk et al or Kumar et al. [6,7]. Besides the SSI, a dynamic stiffness index of medial elastic compression stockings was defined [8].

Liu et al. observed that higher pressures will be reached with increasing a fabric’s bending rigidity [9]. They conclude that thick stocking garments can apply higher amounts of pressure than thin stockings. Liu et al. considered only the fabric elasticity because the bandage’s other properties, measured during the test, depended on subjects themselves (such as body posture, muscle density) [9].

Suehiro et al. conducted a study to evaluate an elastic multilayer bandage’s interface pressure and stiffness [10]. They found that both the parameters increased linearly for up to five bandages, so the bandages’ overlapping increased the stiffness and the friction [10].

Several standards define elastic or inelastic bandages and their properties, but they refer mostly to compression therapy. The main categories of compression bandage elasticity are defined by the standard DIN 61632 as the percent strain of the material following the application of a force of 10 N per centimeter bandage width [11]. These categories are:

(a) Rigid or inelastic: a maximal stretch of 0–10%;
(b) Short-stretch: a maximal stretch of 10–100%;
(c) Long-stretch or elastic: maximal stretch more than 100%.

The classification is technically adequate, but the maximal strains are unlikely to be reached during bandaging, thereby reducing the classification’s value in practical terms.

Partsch et al. classified bandages, carrying out experiments on 10 legs of volunteers with bandages (with different elastic properties) [2]. Partsch et al. proposed another classification [2]:

(a) Rigid or inelastic: a practical stretch of 0–10%;
(b) Short-stretch: a practical stretch of 20–50%;
(c) Long-stretch or elastic: a practical stretch of 40–120%.

Partsch et al. assessed the dynamic modulus (given in N/% stretch) for each category and proposed a classification based on this parameter. The modulus is given by the slope of the load strain curve at the force level of 1 N/cm width:

(a) Rigid: dynamic modulus higher than 30 N/% stretch;
(b) Short-stretch: dynamic modulus higher than 0.3 N/% stretch;
(c) Long-stretch: dynamic modulus lower than 0.3 N/% stretch.

The classification proposed by Partsch et al. is used only for the single bandage components and not for the multilayer compression bandage systems [2].

The RAL-GZ 387/1 gives the quality and test specifications for medical compression hosiery [12]. One of the tests regards the extensibility in longitudinal and traverse directions. The standard specifies the dimensions of the sample: length of 150 mm and width of 50 mm. A load of 50 N is applied within 30 s in order to assess bandage strain.

The above-mentioned standards refer only to bandages with a single component. For a multilayer bandage, Al Khaburi et al. presented a study about the effect of multilayer bandages on the interface pressure applied by compression bandages [13]. To validate the bandage, a test to assess the tension–strain interconnection for the bandage was carried out. They measured the bandage’s tension while it was extended at a constant speed of 100 mm/min and loaded by 100 N.

The ISO 9073-3:1989 specifies the determination of tensile strength and strain in nonwoven textiles using samples with a width of 50 mm and a gauge length (initial length) of 200 mm [14]. Samples must be clamped, and a constant rate of extension of 100 mm/min must be applied. The standard states that the test sample’s strain is determined at the maximum breaking strength and expressed as a percentage of the nominal gauge length.

Adequate patient positioning during a surgical procedure is crucial for the surgeon to approach the patient’s diseased area. Improper patient positioning can cause severe damage, including joint dislocation, nerve impairment, muscular pain and deep tissue injuries to the skin [15]. Patient positioning systems should support high mechanical forces generated, e.g., during dislocation or implantation of hip implants and securing the body against undesired movements [16,17]. Several patient positioning methods have been described in the literature on the specific surgical location and surgical goal [18,19].

In this study, innovative patient position bandages’ mechanical and morphological properties were assessed and compared to two commercially available bandages. The aim was to assess the mechanical properties of the bandage iFix, produced by Interventional systems (iSYS Medizintechnik GmbH, Kitzbühel, Austria), which is used to stabilize patients during an MRI or CT scan. Tensile testing following UNI EN 29073-3 was used to compare the three bandages. Tensile testing following the testing standard UNI EN 29073-3, testing standard UNI EN 29073-2 (wet) and testing standard UNI EN 29073-4 (tear resistance) was used to assess the anisotropic properties of the iFix bandage.

## 2. Materials and Methods

### 2.1. Test Materials

The material properties of iFix (iSYS Medizintechnik GmbH, Kitzbühel, Austria) were assessed and compared to two different bandage materials: Peha-haft (PH) (Hartmann Group, Heidenheim an der Brenz, Germany) and Coban 3M (CO) (3M Health Care, St Paul, MN, USA). All materials were CE-marked and biocompatibility testing was performed by each manufacturer according to the European Medical Devices Directive.

PH is a cohesive conforming bandage composed of 43% viscose, 37% cotton and 20% polyamide. Caused by its texture and micro-structured coating, it is adhesive to itself and has an extensibility of around 85%.

CO is a self-adherent elastic wrap, and it is obtained from natural rubber latex. The wrap is available in sterile and nonsterile versions and used to secure dressings and other devices, to compress or protect wound sites and to immobilize injuries.

**Figure 1:**
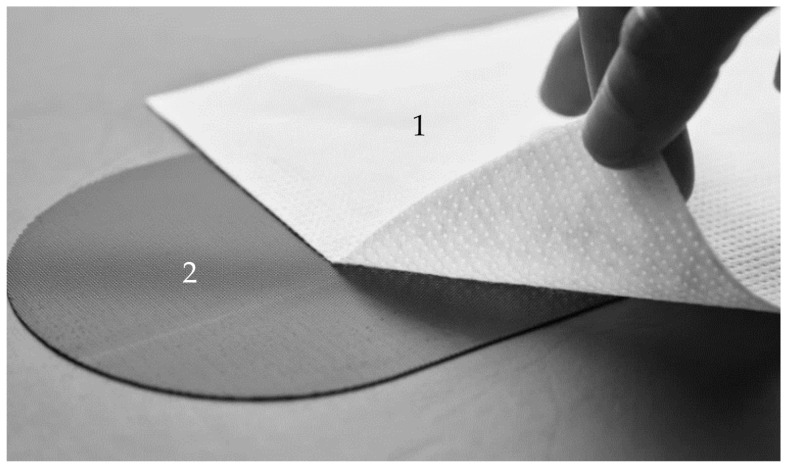
The iFix position system consists of the iFix Fleece (1) and the iFix Patch (2). Adhesive forces on the Patch can fix the Fleece. Image by courtesy of iSYS Medizintechnik GmbH, Kitzbühel, Austria.

iFix is a non-sterile product, intended for patient stabilization over a short period of time, during invasive and non-invasive procedures, by securing the position of the patient’s anatomy with intact skin outside the sterile field. The iFix system is composed of two parts: the iFix Fleece (the bandage) and the iFix Patch (Figure 1). The stretchy fleece is intended for single patient use and, as a single-use component, lowers the risk of cross-contamination. It contours to the patient’s anatomy and secures them in the desired position through its connection to the reusable iFix anchor products. Depending on the table design, an iFix Patch that adheres to the table surface, an iFix Slot Adapter that slides into the channels or an iFix Patch Adapter which connects to standard hook fasteners can be chosen. The micro anchors of the iFix Patches and Adapters strongly secure the iFix Fleece and iFix Spider Fleece to ensure optimal patient stabilization and minimize motion artifacts in the event that an MRI scan is being performed. The iFix Fleece material is polypropylene fleece, nonwoven, has a thickness of 0.83 mm and is used for wrapping the patient. The iFix Patch is composed of extruded polyamide, so by an artificial rubber with humidity and wet resistance. The Patch has a peel strength of 23 N/cm and shear strength of 100 N/cm, and it is used for fixing the iFix Fleece, e.g., fixing it to a surgical table (Figure 1).

### 2.2. Tensile Test

To assess the load and the strain at maximum load for textiles, the strip test was used, where the full width of the sample is gripped in the jaws of an electromechanical material testing machine [20]. The tensile test was carried out to evaluate the load–strain curves of the iFix bandage in all of its directions (longitudinal, transversal, diagonal of 45° and 135°) and to compare the mechanical properties of the iFix bandage, CO and PH in the longitudinal directions. Tensile testing was carried out using an electromechanical material testing machine (Zwicki-Line Z 2.5, maximal load 2.5 kN, 320-kHz sample rate, accuracy ±0.04 N and ±2 µm, Zwick GmbH & Co. KG, Ulm, Germany). All samples had a width of 50 mm and a length of 200 mm. Following a preload of 2 N, a load to failure test in extension with a constant rate of 100 mm/min was applied.

Mean and standard deviation were calculated from the initial length, the maximum load, the load at failure, the strain at maximum load and the strain at failure load obtained from the load–strain curves of ten measurement repetitions. Dynamic modulus (given in N/%stretch) was calculated for each group. This modulus corresponds to the curve’s slope at the force level of 1 N/cm width.

### 2.3. Grab Test

The grab testing method was used for determining the tensile strength in wet samples. The samples of size 100 (width) × 150 (length) mm were clamped only partially using two rectangular jaws with dimensions 25 mm × 25 mm, located centrally on half-width and 12.5 mm away from the long side. Samples were immersed into distilled water for 5 s and then left for 5 s on a PVC support before being clamped and positioned on the testing machine. Five measurement repetitions were carried out using the above-mentioned electromechanical material testing machine at a constant speed of 100 mm/min, following the UNI EN 29073-2 standard [21]. Mean and standard deviation were calculated from the initial length, the maximum load, the load at failure, the strain at maximum load and the strain at failure load obtained from the load–strain curves of five measurement repetitions and used for comparison between iFix in the longitudinal direction (iFix 0°) and iFix in transversal direction (iFix 90°).

### 2.4. Tear Resistance Test

Tear resistance was determined using a tensile test described by UNI EN 29073-4 standard with samples having an initial cut of 10 mm. Samples sized 50 (width) × 230 (length) mm were clamped with jaws at a 30° angle to induce a sequential break of the wires under tensional load. Six measurement repetitions were performed using the previously mentioned testing machine at a constant speed of 100 mm/min, with the samples clamped at its extremities with a 30° angle. Mean and standard deviation were calculated from the initial length, the maximum load, the load at failure, the strain at maximum load and the strain at failure load obtained from the load–strain curves and used for comparison between iFix in the longitudinal direction (iFix 0°) and iFix in transversal direction (iFix 90°). Six measurement repetitions of IFIX in the longitudinal direction (IFix 0°) were compared to PH.

### 2.5. Morphology Assessment Using Scanning Electron Microscopy (SEM)

Samples of the textiles with square form and dimensions of 1 cm^2^ were obtained and fixed on aluminum pins using adhesive Leit-C (Göcke, Plang GmbH, Wetzlar, Germany). Before the examination in the SEM, the samples were coated with a conductive layer of gold (Agar Sputter Coater, Agar Scientific Ltd., Stansted, UK) for 45 s with a sputtering current of 30 mA/mbar. The morphology of the four different materials (iFix fleece, I iFix FIX patch, CO and PH) was analyzed by scanning electron microscopy (SEM, JSM-6010LV, JEOL GmbH, Freising, Germany). For each sample, three images were taken with resolutions of 500 μm, 200 μm and 100 μm at 20 kV using an SEM.

### 2.6. Determination of the Mass of the Fabrics

Before carrying out the mechanical tests, the samples were weighed using a high-precision balance (SI-603, balance precision: 0.001 g, Denver Instrument, Bohemia, NY, USA). Mass was reported as mean and standard deviation for each group.

### 2.7. Statistical Analysis

Shapiro–Wilk test was carried out to assess normal distribution. A *p* < 0.05 was considered as statistically significant in all statistical analyses carried out. One-way ANOVA with Tukey’s multiple-comparison test was used to compare differences between groups with Graph Pad Prism 8.0.1 software (GraphPad Software, Inc., La Jolla, CA, USA). Two-sided *p*-values (*p* < 0.033 *; *p* < 0.002 **; *p* < 0.002 ***, *p* < 0.0001 ****) were considered statistically significant. T-test was used for assessing differences between the longitudinal and transverse orientation of iFix when carrying out grab test and tear resistance test. Graphical representations were made with Graph Pad Prism (GraphPad Software, Inc., La Jolla, CA, USA).

## 3. Results

### 3.1. Comparison of Different Bandages (Tensile Test)

**Figure 2:**
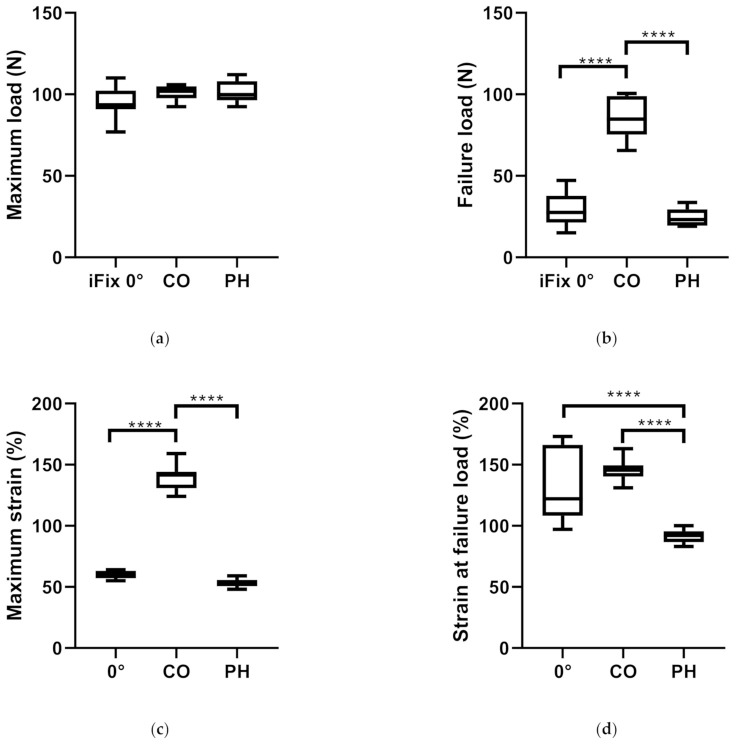
Boxplots showing mean and standard deviation (box) as well as range (whiskers) of the three groups under investigation, iFix, CO and PH: (**a**) Comparison of the maximum load; (**b**) failure load; (**c**) maximum strain; (**d**) strain at failure load. (*p* < 0.033 *; *p* < 0.002 **; *p* < 0.002 ***, *p* < 0.0001 ****).

All measurements showed a normal distribution (*p* < 0.05). No statistically significant difference could be found for the maximum load between PH, iFix and CO (*p* = 0.1104) (Figure 2a). At failure load, a statistically significant higher failure load could be found for CO in comparison to PH (*p* < 0.0001) and in comparison to iFix (*p* < 0.0001). No statistically significant difference could be found between PH and iFix (*p* = 0.5928) at failure load (Figure 2b). CO also showed a significantly higher maximum strain than PH (*p* < 0.001) and iFix (*p* < 0.001). No statistically significant difference could be found for the maximum strain between PH and iFix (*p* = 0.0827) (Figure 2c). When considering the strain at failure load, iFix showed a statistically higher value than PH (*p* < 0.001), and CO showed a statistically higher value than PH (*p* < 0.001). No difference could be found between iFix and CO (*p* = 0.3043) for the strain at failure load (Figure 2d).

With the mean values of the maximum load and the strain, the dynamic modulus was calculated, resulting in a dynamic modulus of 0.72 N/% stretch for CO, 1.94 N/%stretch for PH and 1.64 N/% stretch for iFix. PH dynamic modulus is higher than CO and iFix. The CO dynamic modulus is 63% lower than the PH, and iFix dynamic modulus is 15% lower than the PH. PH and iFix are more rigid than CO, because of their higher dynamic moduli (Table 1).

Using the grab test, a statistically significant higher maximum load was found for iFix in comparison to PH (*p* < 0.0001) and in comparison to CO (*p* < 0.0001) (Table 2). CO showed a statistically significant higher maximum load than PH (*p* = 0.0042). No difference between the three groups could be found for the failure load (*p* = 0.1251). CO showed a statistically significant higher maximum strain than iFix (*p* = 0.0085) and also in comparison to PH (*p* = 0.0116). No difference could be found for the maximum strain between iFix and PH (*p >* 0.05). A statistically significant higher strain at failure could be observed for CO in comparison to iFix (*p* = 0.0271) while no difference could be seen between PH and iFix (*p* = 0.3626) and between PH and CO (*p* = 0.1281) (Table 2).

Using the tear resistance test, a statistically significant difference in higher maximum load could be found for PH in comparison to the iFix bandage in the longitudinal direction (*p* = 0.0141, Table 3). PH showed also a higher maximum load in comparison to CO (*p* = 0.0008), while no difference could be found between iFix and CO (*p* = 0.3359). When considering the maximum strain, a statistically significant higher value could be found for PH in comparison to iFix (*p* < 0.0001) and in comparison to CO (*p* < 0.0001). No difference could be found for the maximum strain between iFix and CO (*p* = 0.8682) when using the tear resistance test (Table 3).

### 3.2. Comparison of Different Orientations of iFix Bandage

**Figure 3:**
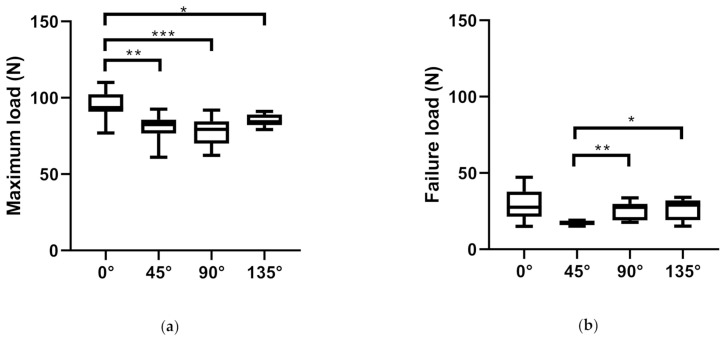

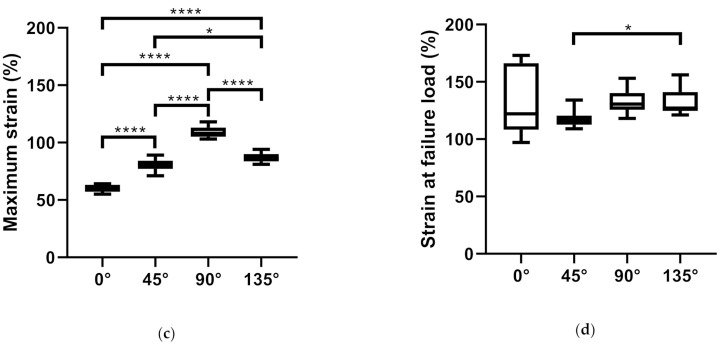
Boxplots showing mean and standard deviation (box) as well as range (whiskers) of the four different orientations of iFix (iFix 0°, iFix 45°, iFix 90° and iFix 135°). (**a**) Comparison of the maximum load; (**b**) failure load; (**c**) strain at maximum load; (**d**) strain at failure load. (*p* < 0.033 *; *p* < 0.002 **; *p* < 0.002 ***, *p* < 0.0001 ****).

When comparing iFix in several orientations (0°, 45°, 90°, 135°) using the tensile test, a statistically significant higher maximum load could be found for iFix 0° in comparison to iFix 45° (*p* = 0.0021), to iFix 90° (*p* = 0.0002) and to iFix 135° (*p* = 0.0358) (Figure 3a). At the failure load, a statistically significant lower value could be found for iFix 45° in comparison to iFix 90° (*p* = 0.0053) and iFix 135° (*p* = 0.0201) (Figure 3b). Considering the strain at maximum load assessed in the tensile test, the highest value could be observed for iFix 90° and the lowest for iFix 0°. iFix 135° showed a statistically significant higher strain at maximum load than iFix 45° (*p* = 0.0245). All other comparisons for strain at maximum load were statistically significant (*p* < 0.0001) (Figure 3c). Assessing the strain at failure load, a statistically significantly higher value could be observed for iFix 135° in comparison to iFix 45° (*p* = 0.0395) (Figure 3d).

The dynamic modulus was calculated, resulting in 1.64 N/%stretch for the longitudinal direction, 0.71 N/%stretch for the transversal direction, 1.03 N/%stretch for iFix 45° and 0.98 for iFix 135°. In the longitudinal orientation, the dynamic modulus is 130% higher than in transverse orientation, 59% higher than in diagonal at 45° and 67% higher than in diagonal at 135°. The dynamic modulus changes with the orientation for the iFix, which is more rigid in the longitudinal than the other orientations (Table 1).

### 3.3. Comparison of Grab and Tear Test of iFix Bandage

A statistically significant higher maximum load (*p* = 0.0006) was measured for iFix in the transversal direction (iFix 90°) in comparison to iFix in the longitudinal direction (iFix 0°) when using the grab test. A statistically significant higher strain at maximum load was observed for iFix 90° in comparison to iFix 0° (*p* < 0.0001). No statistically significant difference could be found for failure load (*p* = 0.1424) or for strain at failure load (*p* = 0.8125) between both groups (Table 2).

A statistically significantly higher value could be found for the maximum load (*p* = 0.0217), for failure load (*p* = 0.0209), for strain at maximum load (*p* < 0.0001) and for strain at failure load (*p* = 0.0002) in the transversal direction (iFix 90°) in comparison to the longitudinal direction (iFix 0°) using the tear resistance test (Table 3).

**Figure 4:**
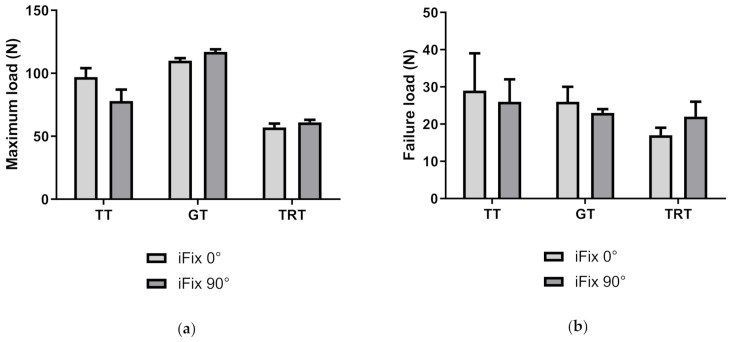

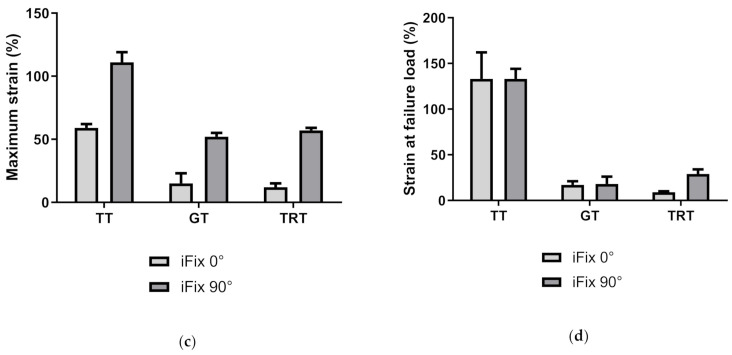
Mean and standard deviation of iFix in the longitudinal direction are shown for the three different test procedures (tensile test TT, grab test GT, and tear resistance test TRT). (**a**) Comparison of the maximum load; (**b**) failure load; (**c**) strain at maximum load; (**d**) strain at failure load.

In Figure 4, the four measurement parameters are reported for the three different test setups to describe the behavior of iFix in dry conditions (tensile test), in wet conditions (grab test) and with the presence of a defect (tear resistance test).

**Figure 5:**
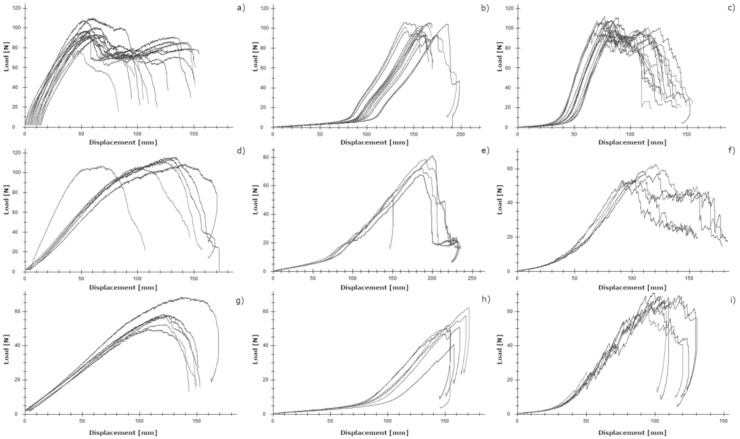
Load–displacement curves of (**a**) tensile test of iFix, (**b**) tensile test of CO, (**c**) tensile test of PH, (**d**) grab test of iFix, (**e**) grab test of CO, (**f**) grab test of PH, (**g**) tear test of iFix, (**h**) tear test of CO and (**i**) tear test of PH.

In Figure 5, the load–displacement curves for the three different test setups are shown to describe the behavior of iFix, CO and PH in dry conditions (tensile test), in wet conditions (grab test) and with the presence of a defect (tear resistance test). From the load–displacement curves, the similar behavior of the three materials can be observed with the three different test setups. iFix, however, shows a more rigid behavior (steeper initial slope) than CO and PH. CO and PH shows a more elastic behavior as, at the initial phase of the curve, the slope is not as steep as in iFix. Especially after reaching the maximum load, a sawtooth pattern was observed for PH, which corresponds to the sequential breaking of the fibers. In CO, maximum load was close to the failure load, while the other two materials, iFix and PH, did not break instantaneously as with CO.

### 3.4. Surface Characterization of Different Assessed Bandages

**Figure 6:**
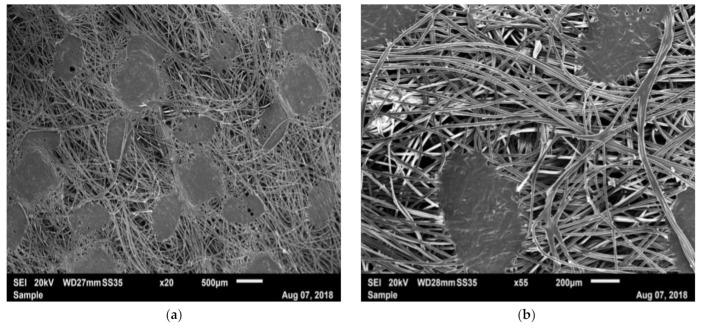

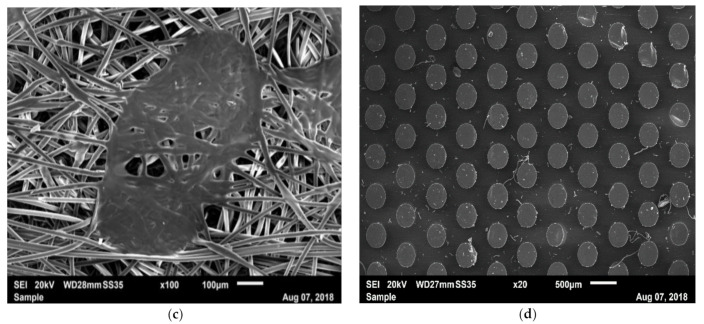
SEM image of iFix with: (**a**) iFix Fleece, ×20 magnification; (**b**) iFix Fleece, ×55 magnification; (**c**) iFix Fleece, ×100 magnification; (**d**) iFix Patch, ×20 magnification.

The iFix bandage presents fibers in all directions, but one direction was predominant: the longitudinal. This was observed in the image with a resolution of 100 μm (Figure 6a), where a higher number of fibers were orientated longitudinally. Fibers were melted together and created structures with an elliptical shape (Figure 6b,c). Instead, the iFix patch showed a stable structure with cylindrical pins where the Fleece could adhere and fix together (Figure 6d). Adhesion of the iFix fleece was obtained only when used in combination with the iFix patch, using the cylindrical pins as anchor points where the fibers could attach.

**Figure 7:**
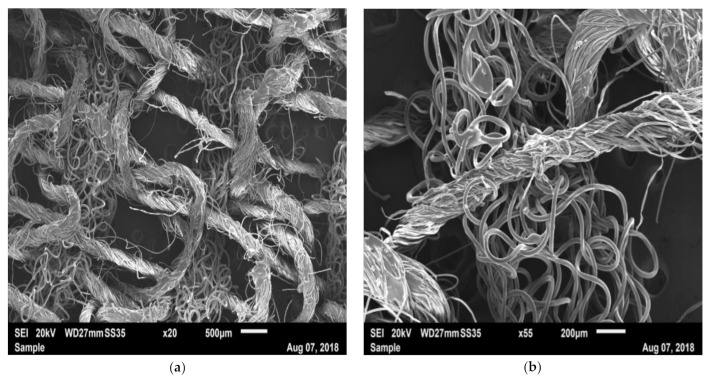

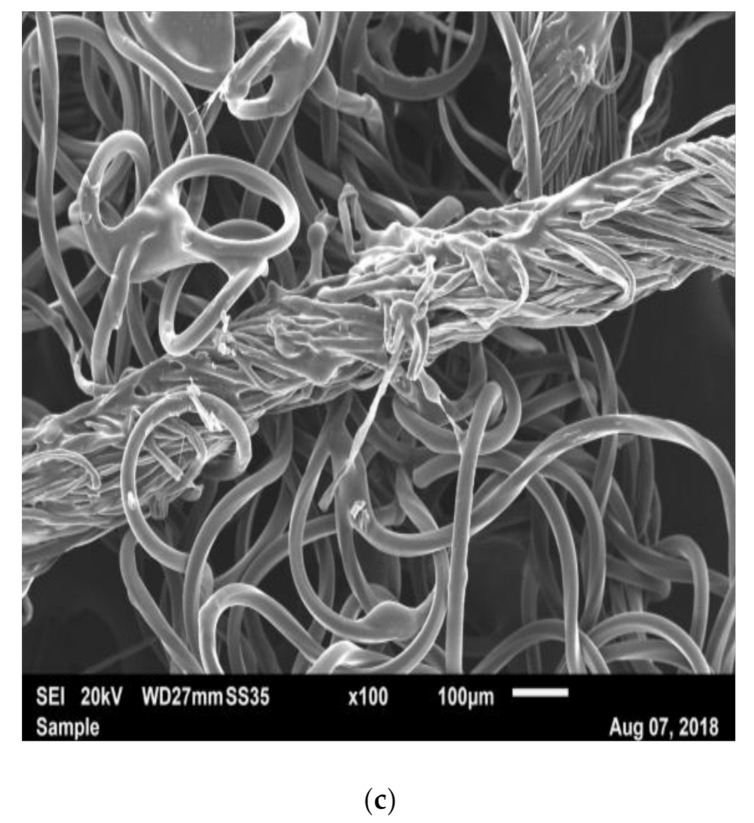
SEM image of PH with: (**a**) ×20 magnification; (**b**) ×55 magnification; (**c**) ×100 magnification.

PH has a structure that differs morphologically from iFix. Two kinds of filaments were observed: the basic structures were entwined fibers, which, at a larger scale, formed a rope and a net, giving the main structure and primary mechanical resistance of the bandage (Figure 7a–c). Between the ropes, some irregular fibers with smaller diameters formed interconnections between the net structures (Figure 7a–c). Woven or knitted structures could be observed with straight longitudinal structures (weft) and fibers which passed over and under the longitudinal structure (warp). From the SEM images, it cannot be deduced whether knots were formed around the warp structure.

**Figure 8:**
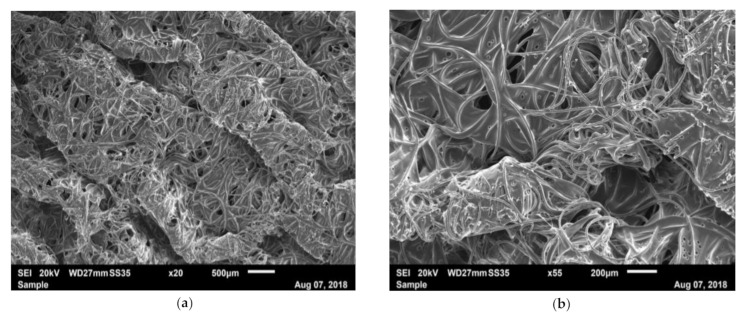

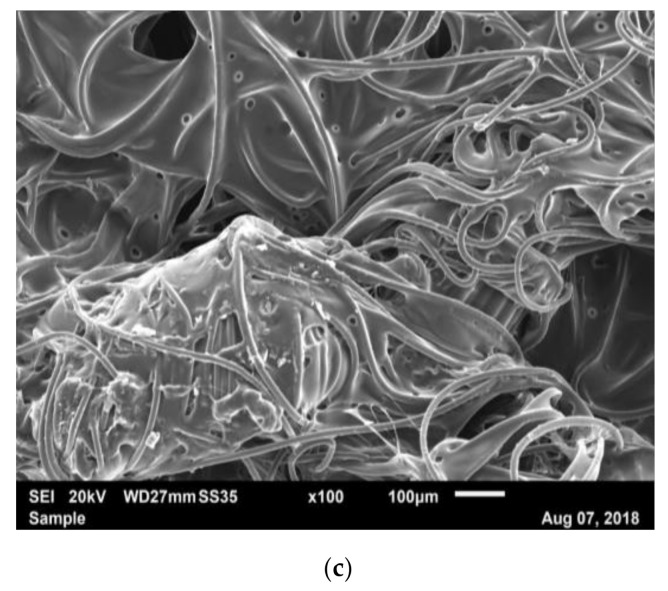
SEM image of CO with: (**a**) ×20 magnification; (**b**) ×55 magnification; (**c**) ×100 magnification.

CO bandage is an elastic bandage with similar properties to PH. CO has shown morphologically a very similar structure to PH, with curvy fibers orientated randomly (Figure 8a–c). The fibers are surrounded by a connecting matrix, which forms a binding structure (Figure 8a–c). The binding structure conceals the woven structures. Strong longitudinal fibers can be observed but the warp structure is not clearly visible in the SEM images. The fibers are connected to each other by a binding material which fills the gaps and presumably provides additional mechanical support.

### 3.5. Mass Measurement of the Three Matierals

Measuring the mass of the three different materials under investigation, it could be observed that CO was almost twice as heavy as iFix and PH (Table 4). The mass of iFix and PH was comparable.

## 4. Discussion

The mechanical properties of the iFix bandage were assessed and compared with CO and PH. Both are available in sterile versions and are used for patient fixation. Morphological analysis using SEM imaging showed different conformations between iFix, CO and PH. CO and PH showed very similar structures; they presented curly fibers oriented in the transverse and longitudinal orientation, forming a net structure with some smaller interconnecting fibers, which act as a linking or filling material. In PH, the smaller fibers may be responsible for the adhering effect when two layers of bandages are placed above each other, as the smaller fibers may create connections between each other in several layers. Especially in CO, a matrix of filling material was observed, which connects the single fibers. It seems that the fibers are responsible for the bandage’s mechanical resistance, while the matrix acts as a filling material. Isotropy was not assessed in the tensile test for PH and CO. However, due to the woven structure of both materials, anisotropy with the main axis in the longitudinal direction can be expected. iFix instead presented with fibers orientated with no main direction; they are melted together in a regular pattern. These points can be considered as tensional nodes; they give rigidity, and they are gripping points for the adhesion between the Patch and the Fleece. Morphologically, it can be deduced that the material might behave anisotropically due to the elliptic-shaped tensional nodes.

Indeed, anisotropy could be observed in the mechanical tests, showing a predominant direction with higher/lower elasticity than in different directions, also under wet conditions (grab test). iFix showed a higher maximum load and failure load in the longitudinal direction than all other directions in tensile testing. In wet samples (grab test), a difference could be observed between the longitudinal and horizontal directions for the maximum load (*p* = 0.0006). However, at failure load, no difference could be observed between different orientations (*p* = 0.1424). When considering the strain at failure load, no difference could be observed between different orientations (*p* = 0.8125), although iFix showed a different strain at maximum load, which was higher in the horizontal orientation than in the longitudinal when using the grab test (*p* < 0.0001).

With a 1-cm incision as a starting point for the tear resistance test, a statistically significantly higher value could be found for the maximum load (*p* = 0.0217), for failure load (*p* = 0.0209), for strain at maximum load (*p* < 0.0001) and for strain at failure load (*p* = 0.0002) in the transversal direction (iFix 90°) in comparison to the longitudinal direction (iFix 0°). It can be deduced that iFix shows more elastic behavior in the transversal direction, while it is stiffer in the longitudinal direction.

When comparing iFix to PH and CO, all three materials showed a similar maximum load. From a mechanical perspective, all three can support a similar maximum load. However, CO showed a statistically significant higher failure load than the other two materials under investigation, which adds an additional safety factor. Concerning the strain at maximum load, it could be shown that CO had a statistically significantly higher strain than iFix and PH. iFix and PH did not show any difference. When considering the strain at failure load, no difference could be observed between iFix and CO. Both iFix and CO showed a statistically significant higher strain at failure load when compared to PH. CO can therefore be considered a more elastic material than iFix and PH. iFix was shown to be more rigid, supporting similar loads to PH.

From the load–displacement curves, it could be observed that iFix showed a more rigid behavior (steeper initial slope) than CO and PH. This might be due to the fact that iFix fibers are connected to each other by tensional anchor points, which give a certain rigidity to the structure. CO and PH showed a more elastic behavior as, at the initial phase of the curve, the slope is not as steep as in iFix. Both materials contain mostly fibers connected to each other in the main longitudinal direction. CO behaves as an elastic material which breaks close to the maximum load. PH, instead, after reaching the maximum load, shows a sawtooth pattern, which corresponds to the sequential breaking of the fibers under loading. iFix and PH did not break instantaneously as with CO and can support some additional stretching after maximum load has been reached, although plastic deformation already occurs. This might give an additional safety factor as failure of the bandage might be detected by the user prior to complete rupture of the bandage.

With the measured values from the tensile test, it was possible to classify the three bandages. The standard DIN 61632 [12] iFix can be classified as a short stretch in the longitudinal and the two diagonal orientations and a long stretch in the transverse direction. Following the standard, PH can be classified as short stretch and CO as long stretch. However, if the dynamic modulus is considered as proposed by Partsch et al. [2], all three bandages under investigation can be classified as a short stretch because their modulus is more significant than 0.3 and lower than 30. All three bandages can therefore be considered rigid.

The measured data suggest that the iFix is comparable to existing bandages considering the supported maximum load. In cases where less rigid fixation is needed, CO may be a better choice than iFix. In cases where rigidity is more important, iFix or PH might be a better choice. The bandage orientation is crucial for iFix, as it showed anisotropic mechanical properties with a significant difference between the longitudinal and transversal directions. iFix showed similar properties when tested in dry and wet conditions, although no direct comparison can be made as two different test setups were used.

The study showed the following limitations: a limited number of repetitions were carried out. However, the number of repetitions follows the requirements of the standards described in Section 2. All tests were carried out in vitro. Therefore, material behavior might vary when applied in surgical settings. Mechanical properties in wet conditions were tested with distilled water. Material properties might change when soaked with other liquids. The morphological assessment was limited to small samples of all three materials. The orientation of the fibers and their composition might change in other areas, which were not investigated.

iFix’s intended use is defined as a short-term patient stabilization device during invasive and non-invasive procedures; it is to be used on intact skin by securing the position of the patient’s anatomy with intact skin outside the sterile field. The stretchy fleece is intended for single patient use and, as a single-use component, it lowers the risk of cross-contamination. It is able to contour to the patient’s anatomy and secures them in the desired position through its connection to the reusable iFix anchor devices. In orthopedic and trauma surgery, it might be an alternative to existing body belts to fix the patient to the surgical table. Belts have the disadvantage of applying local pressure to the skin, which may result in patient harm. iFix can be shaped to patient-specific sheets and be adjusted to the patient’s specific anatomy. Local pressure to the skin may be avoided by using large sheets that are still able to hold the patient in their specific location. iFix Fleece can be used as face masks used to fix the head of the patient by leaving the eyes, nose and mouth unexposed. The application is therefore different from CO and PH as it is adherent only to the iFix Patch products. CO and PH are self-adherent bandages which have to be applied in a double layer to be fixed. They are particularly useful for extremities, although iFix might be used as well as a single layer bandage.

## 5. Conclusions

All three materials investigated in this study supported a similar maximum load. In cases where the rigid fixation of patient limbs or torso is necessary, the authors recommend the usage of iFix or PH, while CO should be used when a higher elasticity is required. iFix showed similar properties in dry and wet conditions; however, the user should be aware of its anisotropic properties when applied as a fixation system for supporting the limbs or torso in a surgical environment. In vivo studies should be carried out to prove the safety of iFix in a surgical environment before its usage.

## Figures and Tables

**Table 1:** Mean and standard deviation of the assessed parameters of the tensile test for iFix in the four different orientations (iFix 0°, iFix 45°, iFix 90° and iFix 135°), CO and PH out of N = 10 measurements.

**Parameter**	**iFix 0°**	**iFix 45°**	**iFix 90°**	**iFix 135°**	**CO**	**PH**
Maximum load (N)	97 (7)	81 (9)	78 (9)	85 (4)	101 (4)	102 (7)
Failure load (N)	29 (10)	18 (5)	26 (6)	27 (7)	86 (13)	25 (5)
Strain at maximum load (%)	59 (3)	81 (5)	111 (8)	86 (6)	139 (10)	53 (4)
Strain at failure load (%)	133 (29)	120 (10)	133 (11)	137 (19)	145 (9)	82 (29)
Dynamic modulus (N/%stretch)	1.64	1.03	0.71	0.98	0.72	1.94

**Table 2:** Mean and standard deviation of the assessed parameters of the grab test for iFix in the two different orientations (iFix 0° and iFix 90° °) out of N = 5 measurements, including *p*-values from the T-test.

Parameter	iFix 0°	iFix 90°	CO	PH
Maximum load (N)	110 (4)	117 (5)	71 (9)	55 (5)
Failure load (N)	26 (7)	23 (1)	18 (5)	18 (3)
Strain at maximum load (%)	15 (20)	52 (7)	126 (87)	15 (16)
Strain at failure load (%)	17 (5)	17 (10)	185 (120)	84 (26)

**Table 3:** Mean and standard deviation of the assessed parameters of the tear resistance test for iFix in the two different orientations (iFix 0° and iFix 90°) out of N = 6 measurements including *p*-values from the T-test.

Parameter	iFix 0°	iFix 90°	CO	PH
Maximum load (N)	57 (6)	61 (5)	52 (7)	68 (3)
Failure load (N)	17 (6)	22 (4)	-	-
Strain at maximum load (%)	12 (6)	55 (4)	14 (9)	42 (7)
Strain at failure load (%)	9 (3)	29 (12)	-	-

**Table 4:** Mean and standard deviation of the mass of the samples used for tensile, tear resistance and grab testing.

Material	Mass of Samples for Tensile Testing (g)	Mass of Samples for Tear Resistance Testing (g)	Mass of Samples for Grab Testing (g)
iFix 0°	0.56 (0.06)	0.81 (0.06)	1.06 (0.03)
iFix 45°	0.61 (0.03)	-	-
iFix 90°	0.64 (0.06)	0.77 (0.03)	1.08 (0.07)
iFix 135°	0.61 (0.02)	-	-
CO	1.10 (0.27)	1.84 (0.04)	2.39 (0.03)
PH	0.54 (0.04)	0.80 (0.02)	1.04 (0.01)

## Data Availability

Research data will be provided on request by the corresponding author.
